# The Wide Distribution and Change of Target Specificity of R2 Non-LTR Retrotransposons in Animals

**DOI:** 10.1371/journal.pone.0163496

**Published:** 2016-09-23

**Authors:** Kenji K. Kojima, Yosuke Seto, Haruhiko Fujiwara

**Affiliations:** 1 Genetic Information Research Institute, Mountain View, CA, 94043, United States of America; 2 Graduate School of Frontier Sciences, University of Tokyo, Kashiwa, Chiba, 277–8562, Japan; 3 Department of Life Sciences, National Cheng Kung University, Tainan, 701, Taiwan; University of Helsinki, FINLAND

## Abstract

Transposons, or transposable elements, are the major components of genomes in most eukaryotes. Some groups of transposons have developed target specificity that limits the integration sites to a specific nonessential sequence or a genomic region to avoid gene disruption caused by insertion into an essential gene. R2 is one of the most intensively investigated groups of sequence-specific non-LTR retrotransposons and is inserted at a specific site inside of 28S ribosomal RNA (rRNA) genes. R2 is known to be distributed among at least six animal phyla even though its occurrence is reported to be patchy. Here, in order to obtain a more detailed picture of the distribution of R2, we surveyed R2 using both *in silico* screening and degenerate PCR, particularly focusing on actinopterygian fish. We found two families of the R2C lineage from vertebrates, although it has previously only been found in platyhelminthes. We also revealed the apparent movement of insertion sites of a lineage of actinopterygian R2, which was likely concurrent with the acquisition of a 28S rRNA-derived sequence in their 3′ UTR. Outside of actinopterygian fish, we revealed the maintenance of a single R2 lineage in birds; the co-existence of four lineages of R2 in the leafcutter bee *Megachile rotundata*; the first examples of R2 in Ctenophora, Mollusca, and Hemichordata; and two families of R2 showing no target specificity. These findings indicate that R2 is relatively stable and universal, while differences in the distribution and maintenance of R2 lineages probably reflect characteristics of some combination of both R2 lineages and host organisms.

## Introduction

Transposons, or transposable elements, occupy considerable fractions of most eukaryotic genomes [1]. The insertion of transposons into a gene is associated with human genetic diseases and cancers [2]. Some groups of transposons, however, have developed target specificity that limits the integration sites to a specific sequence or a genomic region to avoid gene disruption. Although target specificity is reported in LTR retrotransposons and DNA transposons as well [3,4], non-LTR retrotransposons contain the widest variety of target-specific families. Their targets include almost all types of repetitive sequences both functional and non-functional: ribosomal RNA (rRNA) genes, transfer RNA (tRNA) genes, small nuclear RNA (snRNA) genes, microsatellites, telomeric repeats, and transposons [5–10].

R2 is one of the most intensively investigated groups of sequence-specific non-LTR retrotransposons. R2 was originally identified as an insertion sequence in the 28S rRNA genes of the fruit fly *Drosophila melanogaster* [11] and the domestic silkworm *Bombyx mori* [12], and was later characterized as a non-LTR retrotransposon [5,13]. R2 is widely distributed in arthropods [14]. Outside of arthropods, R2 was first reported in Chordata in the zebrafish *Danio rerio* and sea squirts *Ciona intestinalis* and *Ciona savignyi* [9]. To date, R2 has been reported in Echinodermata, Platyhelminthes, Nematoda, and Cnidaria, as well as Arthropoda and Chordata [15–17].

Our previous analyses revealed that several lineages of R2 have been maintained for a long time in animals [15,16]. Four clades (supergroups) of R2: R2A, R2B, R2C, and R2D show independent lineages in the phylogenetic tree based on their reverse transcriptase sequences. They have a distinct number and type of zinc-fingers proximal to the reverse transcriptase domain. R2A has three zinc-fingers, two CCHH type and one CCHC type. R2B has two zinc-fingers, one CCHH type and one CCHC type [18]. R2C also has two zinc-fingers although both of them are of the CCHH type. R2D has only one zinc-finger, which is of the CCHH type. These zinc-fingers are responsible for target recognition, and interestingly the contribution of each zinc-finger to target recognition is different between clades [19,20]. These four clades were further classified into 11 total subclades [15].

R2 is inserted at a specific site inside of 28S rRNA genes [15]. It is dependent on the target-specific cleavage by the endonuclease encoded by R2 [21]. The bottom strand (antisense strand) cleavage is strictly determined, while the top strand (sense strand) shows some variations of cleavage sites, which are determined by the target site alterations upon insertion [16]. Two lineages of R2 have changed their target specificity within the array of rRNA genes. R8 from hydra is inserted in 18S rRNA genes while R9 from the rotifer is inserted at another site of 28S rRNA genes [16,22]. No lineage of R2 that has lost its target specificity has yet been identified.

Here, we surveyed R2 using both *in silico* screening and degenerate PCR. Of interest is the distribution and evolution of R2 in vertebrates. No R2 has been identified from mammals, amphibians, or chondrichthyes, while other groups of vertebrates include at least one species that possesses an R2. R2 has been reported from hagfishes [15], cyclostomes [23], actinopterygian fish [9], coelacanth [24], reptiles [15], and birds [17]. However, no systematic survey of R2 in vertebrates has been performed. Another topic is the origin and distribution of R2 in animals. R2 has been found in many animal phyla, but not yet from Mollusca and Annelida. R2 has neither been found outside the animal kingdom.

In this study, we found several independent lineages of R2 in actinopterygian fish, as well as a single lineage in birds. We also report the first R2 families from Ctenophora, Mollusca and Hemichordata. The loss of target specificity in platyhelminthes and the apparent shift of insertion sites in actinopterygian fish are observed.

## Materials and Methods

### *In silico* screening of R2

Genomic sequences of various species were obtained mostly from GenBank, and sequences of known TEs were obtained from Repbase [1] (http://www.girinst.org/repbase).

New R2 non-LTR retrotransposons were identified by repeated Blastn, tBlastn [25] and CENSOR [26] searches using genomic sequences of various animal species available at NCBI Blast (http://blast.ncbi.nlm.nih.gov/Blast.cgi) and the UCSC Genome Browser (https://genome.ucsc.edu) websites with representatives of each R2 clade (R2A, B, C and D) as queries. To characterize the 5′ ends of R2 from birds, we used Blastn with the 5′ terminal sequences of several R2 families and 5′ flanking 28S rRNA gene sequences. To characterize the 3′ ends of R2 from birds, we used Blastn with the 3′ terminal sequence of *R2-1_TG* and the 3′ flanking 28S rRNA gene sequence. The classification was initially done by RTclass1 [27] and finally determined by phylogenetic analysis. The consensus sequences were derived using the majority rule applied to the corresponding set of multiple aligned copies of retrotransposons. All R2 sequences save for the 3′ end short fragment sequences were named following the systematic nomenclature implemented in Repbase and were submitted to Repbase [1] (http://www.girinst.org/repbase).

### Characterization of R2 from genomic DNAs

Genomic DNA used for screening is shown in S1 Table. Genomic DNA was kindly provided by Dr. H. Mitani and Dr. S. Oda of U. Tokyo for four medaka species, by Dr. M. Nishida of U. Tokyo for other fishes, and by M. Park of U. Tokyo for reptiles. Tissue samples of salamander were kindly provided by Dr. T. Michiue, and tissues of other amphibians by Dr. M. Taira of the U. Tokyo.

To amplify R2 elements, four primers (R2IF1: 5′-AAGCARGGNGAYCCNCTNTC-3′, R2IIF1: 5′-GTNAARCARGGNGAYCCNCT-3′, R2IF2: 5′-GCYYTRGCGTTYGCNGAYGA-3′, R2IIF2: 5′-CTNGCNTTYGCNGAYGAYYT-3′) were designed from the highly conserved region of the RT domain. Four primers were also designed from the downstream 28S rRNA genes (28S_R-198: 5′- GCCTCCCACTTATYCTACACC-3′, 28S_R-147: 5′- GTCAAGCTCAACAGGGTCTTCT-3′, 28S_R-B: 5′-ATCCATTCATGCGCGTCACT-3′, 28S_R-A: 5′-TAGATGACGAGGCATTTGGC-3′). Using the R2 primer and 28S primer pair, PCR was performed with Ex-Taq (TaKaRa) for 35 cycles of 96°C for 1 min, 56°C for 20 s, and 72°C for 2 min. PCR products were cloned into the pGEM-T Easy vector (Promega) and sequenced with ABI PRISM 3130*xl* Genetic Analyzer (PE Applied Biosystems) and BigDye terminator v3.1 cycle sequencing kit (PE Applied Biosystems). Newly identified R2 sequences were deposited in the DNA Data Bank of Japan (DDBJ; http://www.ddbj.nig.ac.jp/index-e.html) and the accession numbers are shown in S1 Table.

### Sequence alignment and phylogenetic analysis

Two types of protein sequence alignments were generated. One (alignment A; 258 sites) is the alignment of the partial RT domains spanning motif 5 to 9 of R2. The second (alignment B; 677 sites) is the alignment spanning RT motif 5 to the C-terminus of R2. In each alignment, sequences including ambiguous residues and/or deletions were excluded. All alignments were generated with the aid of MAFFT with the linsi option [28]. ProtTest was performed at the ProtTest server (http://darwin.uvigo.es/software/prottest2_server.html). Amino acid substitution models were selected based on the Akaike Information Criterion and Bayesian Information Criterion. The models selected were LG+G+F for alignment A and LG+I+G+F for alignment B. Maximum likelihood trees were constructed by PhyML [29] with bootstrap values (100 replicates) using the respective substitution model. For each alignment, all sites or sites selected with the least strict option of Gblocks (129 sites for alignment A and 234 sites for alignment B) were used. As phylogenetic trees based on alignment with Gblocks selection showed weaker statistical support, the phylogenetic trees shown in the figures are based on *all* alignable sites. The phylogenetic trees were drawn with the aid of FigTree 1.3.1 (http://tree.bio.ed.ac.uk/software/figtree/).

## Results

### R2 distribution in invertebrates

**Fig 1:**
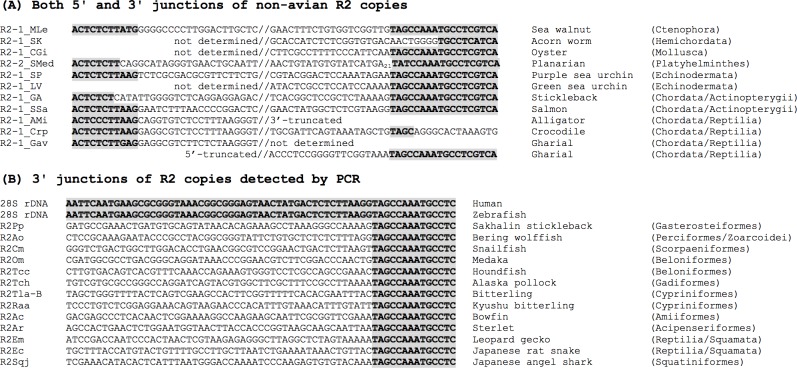
Junction sequences of R2 elements. R2 family name, flanking 28S rRNA gene sequences (28S rDNA) with terminal sequences of R2, common name of the origin, and classification are shown from left to right. 28S rRNA gene sequences are in bold and shaded. (A) Both 5′ and 3′ junctions of non-avian R2 copies. (B) 3′ junctions of R2 copies detected by PCR. Scientific names and common English names of the origins of R2 elements are as follows: R2-1_MLe, *Mnemiopsis leidyi* (sea walnut); R2-1_SK, *Saccoglossus kowalevskii* (acorn worm); R2NS-1_CGi, *Crassostrea gigas* (Pacific oyster); R2-2_SMed, *Schimidtea mediterranea* (planarian); R2-1_SP, *Strongylocentrotus purpuratus* (purple sea urchin); R2-1_LV, *Lythechinus variegatus* (green sea urchin); R2-1_GA, *Gasterosteus aculeatus* (three-spined stickleback); R2-1_SSa, *Salmo salar* (Atlantic salmon); R2-1_AMi, *Alligator mississippiensis* (American alligator); R2-1_Crp, *Crocodilus porosus* (saltwater crocodile); R2-1_Gav, *Gavialis gangeticus* (gharial); R2Pp, *Pungitius pungitius pungitius* (nine-spined stickleback); R2Ao, *Anarhichas orientalis* (Bering wolffish); R2Cm, *Crystallichthys matsushimae* (snailfish); R2Om, *Oryzias melastigma* (marine medaka); R2Tcc, *Tylosurus crocodilus crocodilus* (houndfish); R2Tch, *Theragra chalcogramma* (Alaska pollock); R2Tla-B, *Tanakia lanceolata* (bitterling); R2Raa, *Rhodeus atremius atremius* (Kyushu bitterling); R2Ac, *Amia calva* (bowfin); R2Ar, *Acipenser ruthenus* (sterlet); R2Em, *Eublepharis macularius* (leopard gecko); R2Ec, *Elaphe climacophora* (Japanese rat snake); R2Sqj, *Squatina japonica* (Japanese angel shark).

In addition to the six phyla from which R2 has been previously found, we found R2 families from three phyla: Ctenophora (sea walnut *Mnemiopsis leidyi*), Mollusca (Pacific oyster *Crassostrea gigas*) and Hemichordata (acorn worm *Saccoglossus kowalevskii*) (Fig 1A). We determined the two 3′ junctions for *R2NS-1_CGi* (oyster), which was previously reported as a non-sequence-specific family [30]. Here “*NS*” stands for the Non-sequence-Specific. One of the two copies is adjacent to the 28S rRNA gene, and thus it is not totally non-specific (S1 Fig); we renamed it *R2-1_CGi* (Fig 1A) to reflect this. We also found fragments of R2-like non-LTR retrotransposons from *Priapulus caudatus* (Priapulida), but could not determine the boundaries and thus we are not certain that it is specific for rRNA genes. We have analyzed repetitive sequences from various eukaryotes during the maintenance and expansion of Repbase [1], but have never found any R2 sequences outside animals (data not shown).

### R2 distribution in non-avian vertebrates

**Fig 2:**
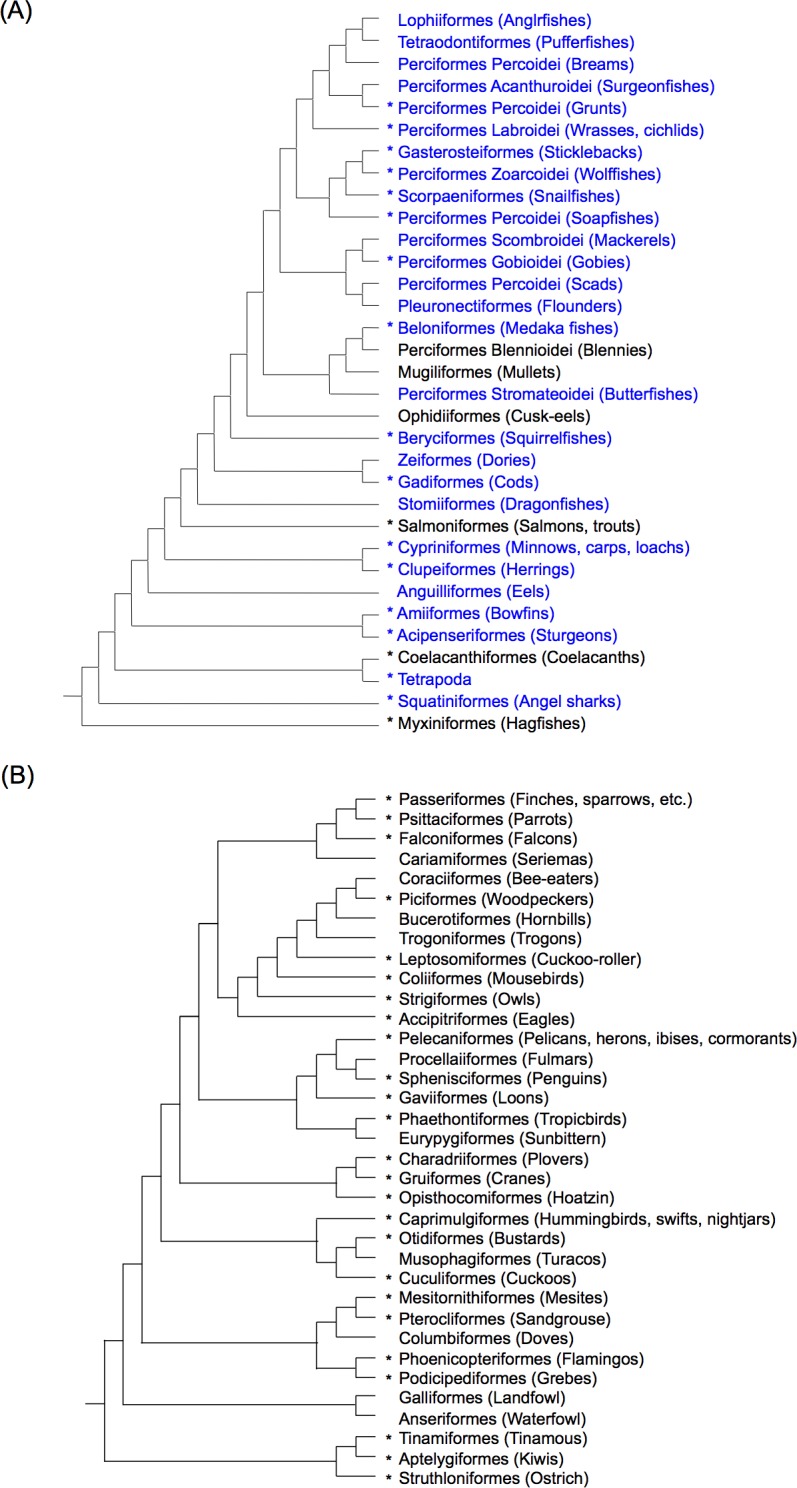
The phylogenetic distributions of R2 in vertebrates and birds. (A) The R2 distribution in vertebrates focusing on actinopterygian fish. (B) R2 distribution in birds. Orders are shown with common names in parentheses. Asterisks indicate the presence of R2 in at least one species. Order names in blue indicate groups that we analyzed by PCR. Perciformes is not monophyletic and thus shown divided. Fish phylogeny is based on [31–33][34,35] while avian phylogeny is based on [36].

Thanks to the recent progress of genome sequencing of Arthropoda and Chordata, we found many R2 families from these two phyla. We focused on the distribution of R2 in fishes because the widest diversity among vertebrates is seen in actinopterygian fishes, although the genome sequences of fish are not yet available from many orders. We used genomic DNA from 34 species in 17 orders of fish (1 order of Chondrichthyes and 16 orders of Actinopterygii), and found R2 from 19 species in 11 orders (Figs 1B and 2A and S1 Table) by PCR. We also characterized R2 *in silico* from three species of fish, stickleback (*R2-1_GA*), platy (*R2-1_XM*), and coelacanth (*R2-1_LCh*). We also characterized R2 from two species of Squamata reptiles (gecko and snake) by PCR. Among non-avian reptiles, turtles and crocodilians have R2 (Fig 1A), although we could not detect R2 from the green anole genome. Moreover, we could not detect R2 from amphibians, whether frogs or salamanders.

### R2 distribution in birds

**Fig 3:**
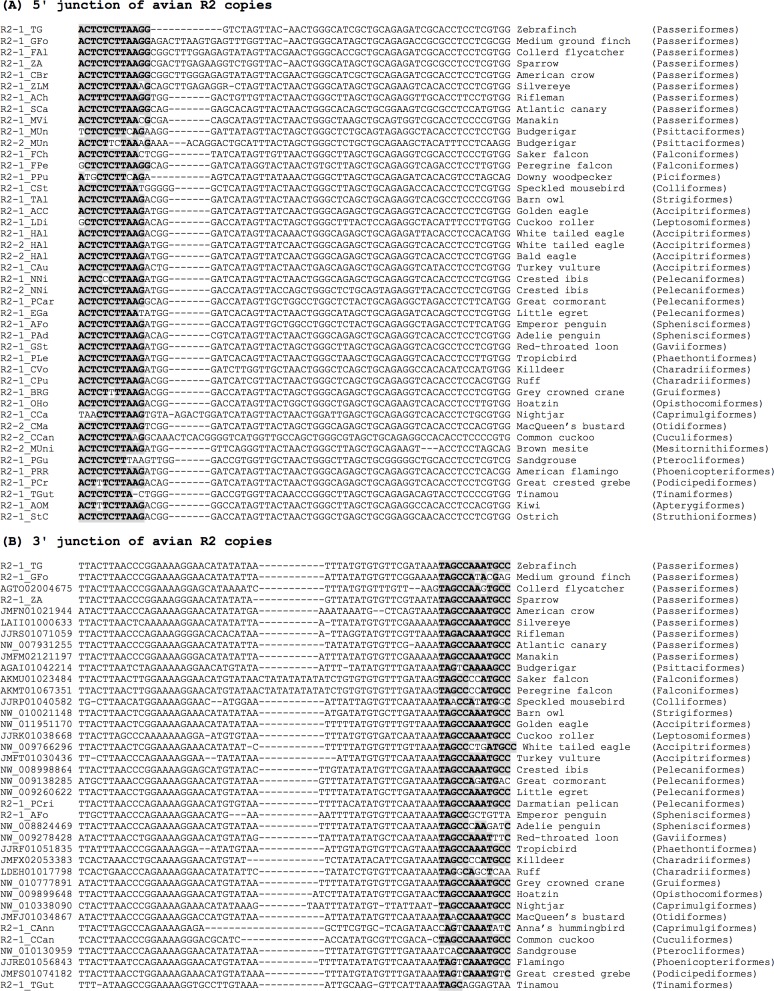
Junction sequences of avian R2 elements. The R2 family name or accession number (if the characterized sequence was short), flanking 28S rRNA gene sequence with terminal sequence of R2, common name of the origin, and classification are shown from left to right. The 28S ribosomal RNA gene sequences are in bold and shaded. (A) 5′ junctions. (B) 3′ junctions. Scientific names and common English names of the origins of R2 elements are as follows: R2-1_TG, *Taeniopygia guttata* (zebrafinch); R2-1_GFo, *Geospiza fortis* (medium ground finch); R2-1_FAl, *Ficedula albicollis* (collared flycatcher); R2-1_ZA, *Zonotrichia albicollis* (white-throated sparrow); R2-1_CBr, *Corvus brachyrhynchos* (American crow); R2-1_ZLM, *Zosterops lateralis melanops* (silvereye); R2-1_ACh, *Acanthisitta chloris* (rifleman); R2-1_SCa, *Serinus canaria* (Atlantic canary); R2-1_MVi, *Manacus vitellinus* (golden-collared manakin); R2-1_MUn and R2-2_MUn, *Melopsittacus undulatus* (budgerigar); R2-1_FCh, *Falco cherrug* (saker falcon); R2-1_FPe, *Falco peregrinus* (peregrine falcon); R2-1_PPu, *Picoides pubescens* (downy woodpecker); R2-1_CSt, *Colius striatus* (speckled mousebird); R2-1_TAl, *Tyto alba* (barn owl); R2-1_ACC, *Aquila chrysaetos canadensis* (golden eagle); R2-1_LDi, *Leptosomus discolor* (cuckoo roller); R2-1_HAl and R2-2_HAl, *Haliaeetus albicilla* (white-tailed eagle) and *H*. *leucocephalus* (bald eagle); R2-1_CAu, *Cathartes aura* (Turkey vulture); R2-1_NNi and R2-2_NNi, *Nipponia nippon* (Asian crested ibis); R2-1_PCar, *Phalacrocorax carbo* (great cormorant); R2-1_EGa, *Egretta garzetta* (little egret); R2-1_AFo, *Aptenodytes forsteri* (emperor penguin); R2-1_PAd, *Pygoscelis adeliae* (Adelie penguin); R2-1_GSt, *Gavia stellata* (red-throated loon); R2-1_PLe, *Phaethon lepturus* (white-tailed tropicbird); R2-1_CVo, *Charadrius vociferus* (killdeer); R2-1_CPu, *Calidris pugnax* (ruff); R2-1_BRG, *Balearica regulorum gibbericeps* (grey crowned crane); R2-1_OHo, *Opisthocomus hoazin* (hoatzin); R2-1_CCa, *Caprimulgus carolinensis* (nightjar); R2-2_CMa, *Chlamydotis macqueenii* (McQueen’s bustard); R2-1_CCan and R2-2_CCan, *Cuculus canorus* (common cuckoo); R2-1_MUni, *Mesitornis unicolor* (brown mesite); R2-1_PGu, *Pterocles gutturalis* (yellow-throated sandgrouse); R2-1_PRR, *Phoenicopterus ruber ruber* (American flamingo); R2-1_PCr, *Podiceps cristatus* (great crested grebe); R2-1_TGut, *Tinamus guttatus* (white-throated tinamou).

Of interest are R2 families from birds. The first R2 family from birds was reported in zebrafinch [17]. Here we screened avian R2 systematically using recently sequenced bird genomes [37]. Initially we characterized several R2 families from birds using tBlastn searches with R2 protein sequences as queries. Based on the obtained avian R2 sequences, we realized that the 5′ end sequences of avian R2 are very conserved (Fig 3A). Then we took this conservation and used it to detect more R2 families by Blastn searches with the junction sequences including both 28S rRNA gene and R2 5′ UTR as queries. We detected more R2 sequences by this method. We were also able to characterize many additional R2 fragments using Blast search with the 3′ junction sequences as queries (Fig 3B).

However, we obtained long protein-coding sequences of R2 only from medium ground finch (*Geospiza fortis*, *R2-1_GFo*), white-throated sparrow (*Zonotrichia albicollis*, *R2-1_ZA*), collared flycatcher (*Ficedula albicollis*, *R2-1_FAl*), and white-throated tinamou (*Tinamus guttatus*, *R2-1_TGut*). Some R2 sequences longer than 3.5 kb with both ends having disrupted open reading frames (ORFs) included R2 from Atlantic canary (*Serinus canaria*, *R2-1_SCa*), white-tailed eagle (*Haliaeetus albicilla*, *R2-1_HAl*), emperor penguin (*Aptenodytes forsteri*, *R2-1_AFo*), and Asian crested ibis (*Nipponia nippon*, *R2-2_NNi*). Most bird R2 copies are severely mutated, although R2 copies flanked by 28S rRNA gene fragments on at least one side are observed in various bird species (Fig 3). Their strong sequence similarity supports the common origin of R2 in birds. There is a tendency for R2 copies from more closely related species to show higher identity. For example, the 5′ 100-bp sequence of *R2-1_TG* from zebrafinch (Passeriformes) shows a 92% identity to that of *R2-1_ZA* from the white-throated sparrow (Passeriformes), 84% to that of *R2-2_HAl* from the white-tailed eagle (Falconiformes), and 79% to that of *R2-1_TGut* from the tinamou (Tinamiformes). However, this trend is not always clear; the 5′ 100-bp sequence of *R2-1_SCa* from the canary (Passeriformes) is only 76% identical to that of *R2-1_TG*. This may be partly due to the accumulated mutations since the inactivation of *R2-1_SCa*.

Finally, we found R2 copies from 25 orders spanning a wide range of bird lineages (Fig 2B). R2 is distributed among almost all of the major groups of birds, except Galloanseres (chickens and ducks). This is consistent with our previous PCR experiments that failed to detect R2 in two Galloanseres species (mallard and chicken) [9]. We found two fragments of R2 copies in budgerigar, which was one of the two other species for which we could not amplify R2 by PCR. One copy in AGAI01067519 (*R2-1_MUn*) is severely truncated and corresponds to the 5′ ~730-bp sequences of *R2-1_AFo* and *R2-2_NNi* (emperor penguin and Asian crested ibis). The other copy (*R2-2_MUn*) is an internally deleted copy, which shows similarity to the 4–517, 604–726, and 4370–4700 regions of *R2-2_NNi*. In both cases, the 5′ ends are flanked by a sequence with weak similarity to 28S rRNA genes. This could explain why we could not amplify R2 fragments by PCR in budgerigar.

### Phylogenetic analysis and distribution of R2

**Fig 4:**
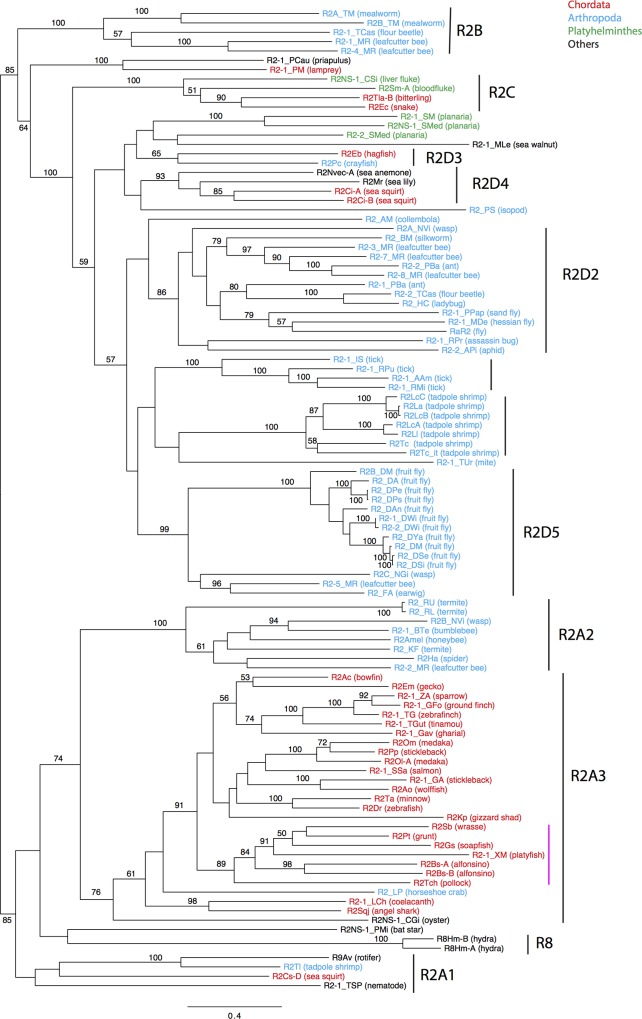
A phylogenetic tree of R2 families based on the protein alignment from motif 5 of the RT domain to the C-terminus. Bootstrap values above 50% are shown at branches. R2 family names and their origins are shown as leaves. R2 families from Chordata are colored in red, those from Arthropods in blue, those from Platyhelminthes in green and those from other animals in black. Clusters of R2 families that can be assigned to reported subclades are indicated by vertical lines with names whilst clusters not assigned to reported subclades are indicated by vertical lines without names. The magenta bar indicates the cluster having a 28S rRNA gene-like sequence in the 3′ UTR.

We generated two phylogenetic trees of R2 families. One is based on the alignment of the partial RT domain corresponding to motifs 5 to 9 (S2 Fig). The other is based on the alignment of motif 5 to the C-terminus (Fig 4). We note that we could not recover intact ORFs for not a few R2 families due to incomplete sequencing, mutations or truncation, and thus, we could not use them in the phylogenetic analysis. The two trees showed a similar topology. We tried to determine the root using outgroup sequences, but failed to obtain a consistent result. In addition to the clusters of R2 families that can be assigned to reported subclades (vertical bars with names in Fig 4), two new clusters were supported with high statistical significance (vertical bars without names in Fig 4), consistent with the original articles on R2 from ticks, and tadpole shrimps [38,39]. These two clusters can be equivalent to subclades, although their distributions might be narrow. The subclades that include R2 families from more than three phyla are R2A1 (Chordata, Arthropoda, Nematoda), R2A3 (Chordata, Arthropoda, Platyhelminthes, Mollusca), and R2D4 (Cnidaria, Chordata, Echinodermata).

Although R2 from Chordata is seen in six lineages, R2 from jawed vertebrates are seen only in two lineages, R2A3 and R2C. *R2Tla-B* and *R2Ec* belong to the R2C clade. All other retrotransposons belonging to the R2C clade are from trematodes (Platyhelminthes): *R2Sm-A* and *R2Sm-B* from the bloodfluke *Schistosoma mansoni* and *R2NS-1_CSi* from the liver fluke *Clonorchis sinensis*. To exclude the possibility of contamination, we sequenced the 3′ downstream 80-bp sequences from the R2 insertion sites. The sequences downstream of *R2Tla-B* and *R2Ec* are identical to the 28S rRNA genes from vertebrates and not identical to those from *Schistosoma* (S3 Fig). Trematodes are parasites infecting mostly vertebrates. Horizontal transfer of R2C from trematodes to vertebrates cannot be excluded, but the long-term maintenance of R2C since the split of trematodes and vertebrates (protostomes and deuterostomes) is another possibility.

We found only one family of R2 in each vertebrate species we analyzed with the sole exception of alfonsino (*Beryx splendens*), which contains two closely related families (*R2Bs-A* and *R2Bs-B*). However, we may have missed R2 subfamilies or lineages that are difficult to be amplified by PCR with our primer sets. However, the relationships among R2 families from two medaka species (*Oryzias melastigma*, *R2Om* and *Oryzias latipes*, *R2Ol-A*) and from two stickleback species (*Pungitius pungitius pungitius*, *R2Pp* and *Gasterosteus aculeatus*, *R2-1_GA*) indicate the maintenance of two R2 lineages in their ancestral species. Another explanation for these relationships may be the occurrence of horizontal transfer. It is likely that more than one lineage of R2 has been maintained in vertebrates, as reported in arthropods [40,41].

Newly identified R2 families from insects were clustered with reported insect R2 families R2A2, R2B, R2D2, and R2D5. In arthropods, it is more obvious than in vertebrates that several lineages of R2 have been maintained in a single species. The leafcutter bee *Megachile rotundata* is the outlier case in that all four lineages that were found in insects (R2A2, R2B, R2D2, and R2D5) were observed.

The phylogenetic position of *R2-1_MLe* from the sea walnut is of interest since Ctenophore is one basal lineage of animals. Although we could not determine its phylogenetic position with high statistical confidence, its capacity of encoding a protein with a single CCHH zinc finger at the N-terminus indicates that *R2-1_MLe* is inside the R2D clade.

### Non-target specific R2 families

We found two families of R2 that are not specifically inserted into 28S ribosomal RNA genes. One is *R2NS-1_SMed* from the Mediterranean planaria *Schmidtea mediterranea*, belonging to the R2D clade, and the other is *R2NS-1_CSi* from the liver fluke *Clonorchis sinensis*, belonging to the R2C clade. We confirmed their non-specific integration by analyzing their flanking sequences (S1 Fig). Some copies are >98% identical to their respective consensuses, eliminating the possibility that recombination contributed to their apparent lack of sequence specificity. Thus the loss of target specificity has occurred independently in two different families from the R2 lineage.

We could not determine the boundaries of *R2NS-1_PMi* from the bat star *Patiria miniata*, as we did not find rRNA genes in the scaffold sequence (AKZP01104910). At present we have no evidence for the target specificity of *R2NS-1_PMi*, but it is possible that other copies are inserted into 28S rRNA genes.

#### Apparent movement of insertion sites of R2 families

**Fig 5:**
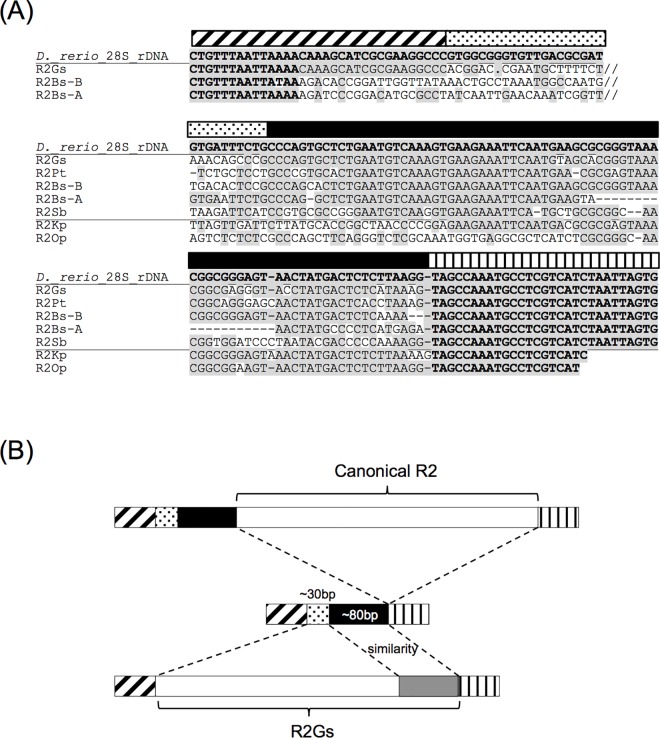
Apparent movement of 5′ insertion sites of R2. (A) Alignment of 5′ and 3′ junction sequences. Nucleotides of 28S rRNA genes are in bold and shaded while nucleotides identical to 28S rRNA genes inside of R2 elements are only shaded. Patterned boxes above the sequences correspond to the regions with the same patterned boxes in (B). *R2Gs*, *R2Pt*, *R2Bs-B*, *R2Bs-A*, and *R2Sb* are phylogenetically closely related and it is likely that their 3′ 28S-like sequences were acquired in their common ancestor. (B) Schematic diagram around the insertion sites of canonical R2 and *R2Gs*. Patterned boxes indicate the regions of 28S rRNA genes. Scientific names and common English names of the origins of R2 elements are as follows: R2Gs, *Grammistes sexlineatus* (goldenstriped soapfish); R2Pt, *Parapristipoma trilineatum* (chicken grunt); R2Bs-A and R2Bs-B, *Beryx splendens* (splendid alfonsino); R2Sb, *Stethojulis bandanesis* (red shoulder wrasse); R2Kp, *Konosirus punctatus* (dotted gizzard shad); R2Op, *Oxyurichthys papuensis* (arrowfin gobies).

Seven R2 families show a similarity to the 28S rRNA gene sequences in their 3′ UTR (Fig 5A). The identity of this region is not as high as that downstream of the canonical insertion site of R2. This indicates that these 28S-like sequences are parts of the 3′ UTR of R2. Among them, five (*R2Gs*, *R2Pt*, *R2Bs-A*, *R2Bs-B*, and *R2Sb*) are phylogenetically closely related (Fig 4, magenta bar). The lineage that includes these five families also includes one more family that was identified from the sequenced genome, *R2-1_XM* from the platyfish *Xiphophorus maculatus*. We also found a fragment sequence of R2 from the Amazon molly *Poecilia formosa* (AYCK01024837). Unfortunately we could not characterize their 3′ termini since all of these sequences were either fragmented or unsequenced, and not flanked with 28S rRNA genes. *R2Tch* does not have a sequence similar to 28S rRNA genes in its 3′ terminus, unlike its sister lineage.

We were able to characterize the 5′ junctions of three of these R2 families (*R2Gs*, *R2Bs-A*, and *R2Bs-B*). Their 5′ junctions are upstream from the canonical insertion site of R2—around 110 bp away in the case of *R2Gs*, and around 130 bp away in the cases of *R2Bs-A* and *R2Bs-B* (Fig 5A). This indicates that upon the insertion of these three families of R2, around 110–130 bps of 28S rRNA gene sequence is replaced by R2 (Fig 5B).

## Discussion

### The distribution of R2

Nine animal phyla (Arthropoda, Chordata, Echinodermata, Hemichordata, Nematoda, Platyhelminthes, Mollusca, Cnidaria, Ctenophora) have maintained at least one of the R2 lineages. Cnidaria and Ctenophora are basal lineages of animals. Four major groups of bilaterian animals, Deuterostoma (Chordata, Echinodermata, and Hemichordata), Ecdysozoa (Arthropoda and Nematoda), Platyzoa (Platyhelminthes) and Lophotrochozoa (Mollusca), all include R2-harboring species. R9, which is a derivative of R2, was reported from Rotifera in Platyzoa [22]. We found an R2-like sequence in Priapulida in Ecdysozoa. It is now clear that R2 is very widely distributed in animals. However, considering sublineages (subclades) of R2, the distribution is apparently patchy. Most of the R2 subclades were reported only from one phylum. We consider that it is partially due to the sampling bias for Arthropoda and Chordata. It should also be mentioned that we may have failed to amplify R2 sequences due to the sequence differences from the primer sets we used and there is a possibility that each R2 lineage may be more widely distributed.

The R2 families from basal groups of animals (Cnidaria and Ctenophore) appear not to be the basal lineages of R2. *R2Nvec-A* belongs to the R2D4 subclade. *R8Hm-A* and *R8Hm-B* are positioned inside of the R2A clade. *R2-1_MLe* is probably a distinct lineage inside of the R2D clade. Their phylogenetic positions indicate that the origin of R2 predates the birth of metazoa. At present, there is no reason to introduce horizontal transfer to explain the distribution and phylogeny of R2, but there is a possibility that ancient horizontal transfer between different metazoan lineages complicated the R2 distribution further. It is noteworthy that even if horizontal transfer has occurred, the very ancient origin of R2 is evident.

In vertebrates, the R2A3 subclade is the dominant lineage, even though some other lineages (R2C, R2D3 and *R2-1_PM*) are also present in some species. The phylogenetic relationships in R2A3 are not always consistent with the host vertebrate phylogeny, and the finding of more than two families of R2 in some fish species or genera indicates that multiple lineages of R2A3 have been maintained in some groups of vertebrates. Considering the presence of multiple lineages of R2 in insects, this is quite likely.

From teleost fishes, we recovered R2 sequences mainly belonging to the R2A3 subclade, flanked with 28S rRNA genes. R2 families characterized from birds in this study also belong to the R2A3 subclade. We could find R2 sequences from most of the bird genomes we analyzed. However, many R2 sequences were disrupted or not yet completely sequenced. Only some R2 copies are intact and encode a full-length protein. Some R2 copies seem full-length but contain disrupted protein-coding regions. It is likely that many R2 sequences were remnants of anciently active R2 elements. It can explain why many avian R2 sequences are flanked with fragmented 28S rRNA gene sequences or non-rRNA gene sequences.

The phylogeny of R2 proteins from birds is consistent with the host phylogeny. The closest relatives of bird R2 sequences are R2 families from crocodilians (*R2-1_Gav* from gharial in Fig 4). This suggests that only a single lineage of R2 is present in birds.

The conservation of the 5′ end sequences of avian R2 appears extraordinary. We could not quantify their conservation in terms of identity, but the 5′ ends of bird R2 appear to be more conserved than those of *Drosophila* R2. It may indicate the importance of the 5′ UTR sequences in R2. It is reported that some R2 families contain an HDV-like ribozyme in their 5′ UTR which contributes to the 5′ processing and translation initiation [42,43]. In *R2Dm* from *Drosophila melanogaster*, the most conserved sequence, CCUCCUCGUGG, is positioned at nucleotides 135–145, while the identical sequence is positioned at nucleotides 37–47 in *R2-1_TG* from zebrafinch. Thus, natural selection for retention of ribozyme function likely contributes to the higher conservation in the 5′ 100-bp sequence of avian R2 than in *Drosophila* R2.

### Change of target specificity of R2

To date, only two lineages in the R2 superclade, R8 and R9, have been reported to have different target specificity from canonical R2 families. Here, we report two clearly non-target-specific R2 families: *R2NS-1_SMed* and *R2NS-1_CSi*. Their phylogenetic positions are distinct, indicating independent loss of target specificity. Since all other clades of non-LTR retrotransposons that show target specificity also include some non-target-specific families, it shows that R2 is not an exception in this facet. The conserved target specificity of R2 is still exceptional. One of the reasons for this maintenance of target specificity is certainly the suitability of its target, rRNA genes, which are highly conserved sequences with high copy numbers. It is unclear why two R2NS families (*R2NS-1_SMed* and *R2NS-1_CSi*) lost their target specificity. The genome of the Mediterranean planaria *S*. *mediterranea* also contains two R2 families showing canonical target specificity (*R2-1_SM*, and *R2-2_SMed*). It is also noteworthy that the genome of *S*. *mediterranea* contains several families of target-specific non-LTR retrotransposons, belonging to the NeSL clade [10]. The targets of three of these families (*LIN9_SM*, *LIN24_SM*, and *LIN26_SM*) are 28S rRNA genes and thus, the loss of target specificity of *R2NS-1_SMed* may be caused by the competition of target 28S rRNA genes with other target-specific non-LTR retrotransposon families. No canonical R2 family has been characterized from the genome of the liver fluke *C*. *sinensis*.

One vertebrate R2 lineage includes families having 28S rRNA gene-derived sequences in their 3′ UTR (Fig 4, magenta bar). This seems related to the apparent movement of 5′ boundaries of R2 insertions upstream compared to those in canonical R2 families (Fig 5). A similar case is observed in *R2Hm-B* [16]. Based on the 3′ junction sequences, *R2Hm-B* appeared to be inserted 15 bp upstream relative to *R2Hm-A*. One interpretation for this is that *R2Hm-B* has a 15-bp sequence identical to 28S rRNA in its 3′ terminus. In the case of families of fish R2, the 3’ UTR sequences are distinct from 28S rRNA genes and thus we can exclude the possibility of the movement of 3′ junctions, which likely correspond to the bottom strand cleavage site. Rather, the situation indicates that a region over 100 bp long of the 28S rRNA gene is replaced by R2 upon integration (Fig 5B). This corresponds to the movement of the top strand cleavage site. Since the top strand cleavage site, unlike the bottom strand cleavage site, is not strictly determined, this is a likely explanation, though further investigation is necessary. The similarity of the 3′ UTR with the 28S rRNA gene may contribute to stabilizing the transposition intermediate through the binding between R2 mRNA and 28S rRNA genes, like other target-specific non-LTR retrotransposons [44,45].

## Supporting Information

S1 Fig Flanking sequences of non-target-specific R2 families. The top 30 hits with 3′ termini in the Censor search are shown. R2 is inserted at “|”. The positions of R2 copies are shown in parentheses. 28S rRNA sequences are in bold.
(PDF)

S2 Fig A phylogenetic tree of R2 families based on the protein alignment from motif 5 to 9 of the RT domain. Bootstrap values above 50% are shown at branches. R2 family names and their origins are shown at leaves. R2 families from Chordata are colored in red, those from Arthropods in blue, those from Platyhelminthes in green and those from other animals in black. Clusters of R2 families that can be assigned to reported subclades are indicated by vertical lines with names and clusters not assigned to reported subclades are indicated by vertical lines but without names.
(PDF)

S3 Fig The downstream 100 bp of *R2Tla-B* and *R2Ec*. Nucleotides identical to the 28S rRNA genes from humans are shown by dots (.).
(PDF)

S1 Table Genomic DNA used for screening. (PDF)
