# Supplementary material for: The Wide Distribution and Change of Target Specificity of R2 Non-LTR Retrotransposons in Animals
Source: PLoS One. 2016 Sep 23;11(9):e0163496. doi: 10.1371/journal.pone.0163496 (PMC5035012; doi:10.1371/journal.pone.0163496)
Supplement: S1 Fig — The top 30 hits with 3′ termini in the Censor search are shown. R2 is inserted at “|”. The positions of R2 copies are shown in parentheses. 28S rRNA sequences are in bold. (PDF) [file pone.0163496.s001.pdf]

S1 Fig

R2-1_CGi (renamed from R2NS-1_CGi)

AFTI01009495_[1040-2536] CTGATTACATTGAAGTCATT|**TAGCCAAATGCCTCGTCATT**
AFTI01001525_[34693-34077] ATATAGTTGCCCAGCTGAAG|TAGACGGACTAGTAATGCGG

R2NS-1_SMed

AAWT01060852_[11519-5624] ATGGGGCGCCGGTTATTCGC|CATAACTTGCAGCATGTGAC
AAWT01034971_[879-6581] ATTTTCACAATCAGTAAATA|CATTTATACTTCTTTTATAA
AAWT01056501_[38986-34390] ATGGTATATATATATTATGT|CATGGCGTTTCGTGTTATTT
AAWT01059403_[14404-10210] CTCGTTGGACCCATGGATCG|AAACCTTGACCAAAAATATA
AAWT01075205_[2857-7059] CAATACCTATGGTTTTTCAC|ATCTTTTTTACTTTTTCTTC
AAWT01013339_[10587-13658] ACGATTCCGCTGCGAAAATG|GGTATATATACCTGTTTTGT
AAWT01024174_[4307-1353] NNNNNNNNNNNNNNNNNNNN|CATGTAATAGTTGTGTATAG
AAWT01030918_[9966-6932] ATAACCTCGACTCCGGAAAT|AAACATTTTATTTTTAATCA
AAWT01072401_[27839-25133] ATGGAATTGATTGATATTGG|CATTGATATGCTTTAATTTT
AAWT01000599_[7466-4902] CCTTTCTGGTGCTGTGACTC|ATTCAAAGTTGCAGTGACCC
AAWT01065092_[13916-16624] TATAATATTTTCAAAAAATT|CAACTTATCAGTTTTCTGTC
AAWT01057482_[6294-3956] AAACGTGATTCAAACGTGAA|CATAACGAGATTGGACCAAA
AAWT01008539_[37632-35310] TAGTAACTCCTCGGCTTGAT|CATAGTCGTTGCTAGAGGCT
AAWT01038751_[615-2798] TGCGTGTTAATTTTATTGCG|CAATGAATTATTCATGAATT
AAWT01015348_[1706-3804] ATTCAAGCTTTCCGTGATTT|TAATAAAAGTTCACTCTTAT
AAWT01056738_[12485-10365] NNNNNNNNNNNNNNNNNNNN|CATTAAAATATTAATAATAA
AAWT01087548_[1589-3564] CTAGTTCAGACGGATGATCA|CATAACGTGATCACCGAGAC
AAWT01018322_[30532-32471] TATATAAAAAATAATTTACA|GCTGAGGAATCTCGCTGAGG
AAWT01055077_[15918-17601] AACTTTTGTTGCAAACCAAG|CATGTGCGACAGATACTGAA
AAWT01022045_[11294-9581] GAATTAGACCGGTAAATTAT|AACAATGAATTAAAACTATT
AAWT01052660_[92844-91218] TGTGGGGTTTGTTGGAGGAA|TTATCATTATTTTTGATGCG
AAWT01049203_[1-1584] NNNNNNNNNNNNNNNNNNNN|AACCTTTGGAATCTTTGATA
AAWT01068947_[21384-19821] GTCCCTCATTTCGTCCCTCA|CTGGCAGTATGTTCTGGAAC
AAWT01032388_[37650-36122] NNNNNNNNNNNNNNNNNNNN|AGTTGCATAATACCTCGATG
AAWT01011910_[6472-5187] GATAGAGTACTACCTATACC|CTACCTATACCGCTATATCA
AAWT01080454_[15462-14178] GATAAAGTACTACCTATACC|CTACCTATACCGCTATATCA
AAWT01071046_[12349-11208] GTCTGCCTATAGGTAAATCC|CATGAGTTAAAAAAATGAGT
AAWT01061248_[4534-3442] TCCTTGCTTAAGTTTTGAAA|CTGAATTACTTCATAATAAT
AAWT01060630_[3598-4823] TTGATAATATTACTATTTAT|AAAATAATTACAATAATAAA
AAWT01002749_[8872-10250] GATAATATTACTATTTATGG|AAAATCATTACAATAATAAA

R2NS-1_CSi

BADR02001492[146176-142575] GTTGTGGATTTACCTTACTA|ATTAAAGGTAAGTCCTGCTT
BADR02000207[128471-132063] AAGTAAAAAATAACAAAATT|AGTAAGTAAGTAAGTAAGTA
BADR02002931[352868-356469] CTGCTCTCGACGCACTTTTC|ATTGGTGTCTCAGAGCTTAC
BADR02003303[1478-5055] AGTAGTAGTAGTAGCAGTGG|GCCGTAAGCCAGCCGATGAC
BADR02001976[63685-67223] TAGTGAATTGTAGTGAATAG|ACGGTGAGTTGCTGCTTTTT
BADR02002036[42182-45740] ATGGGCTCTGCCAAAAACCA|GTATTTGGGTACCACAGTTG
BADR02001385[14211-17149] GTGTTATGAGTAGTATGATT|ATATTATAAAGAGTGGGCGG
BADR02001093[184336-187875] AAAAATATGATAAAAATATG|TTAGATGTCACATGCCACCC
BADR02000094[371104-374619] TGTTTGGAGGTGTAGGCAAC|ATTTTTGTAAGCTAGCACAA
BADR02001160[69223-72792] CGCAAGTATAAAATACAGGC|AATAGACTTAATACACTACT
BADR02002934[228303-231833] ACCAGCATTTCATGAACTGT|ATTGTACAATTAACGGGCTT
BADR02000358[207085-210636] TGATTGCTACGAATAAATTT|GTAAATGACAGTCTCCGAAT
BADR02002508[47672-44161] TGTCGTAAGTAGTGTATAAC|GATGTAAGTAGTGTATAACT
BADR02002930[449232-452488] AGCTGGAATAGACAAATGAT|GTAGTTCGAGTGCACACTCT
BADR02002959[321163-324677] TACTTTGCGAGCTTAGTAAT|AAGATAGAGCGTCACTCACC
BADR02001749[40259-43535] ATATATATATAATATTTCTC|AAAAAAATATAGTCAACTTG
BADR02000342[5289-8806] GGATTATAGCCCTCACCTAC|ATATGAGATCCATATCATGT
BADR02000405[156042-152997] GCAAATGAGTGTATTTCGTT|AATAGAACATACGGCTGACG
BADR02001464[72263-68965] AACTTTTCTAACTTTTTCTC|GACGTTGGAAATTAAACCAT
BADR02000043[655277-651798] ATAACAGTAGGAGCTGTGTG|AATGCTGAAGAGAGCACTCT
BADR02000536[90214-93646] CGGTGCAGCTTCATCTAAAT|TTAGTCAATAAAACATGTCT
BADR02000600[41312-44231] GTTAAGCTGCGTGCATTAAT|GTATTGGCCTTACCTCCCAG
BADR02001831[14598-17016] TGATATACGGCTCCTGAGGA|ATAAGGCGTATATAGGCGAA
BADR02002947[315419-312598] TCGCCGGCACGCACACCCCA|CTGTAGTAGTAGACGAAGTC
BADR02000275[363548-360081] GTGGTGTTGTTTCAAGAAAT|GTATGTTGCTTCAGTGCGAA
BADR02003268[106792-109159] GGGTGGACAAGAGATCCGTA|AAGATAGTGACACACCCCTT
BADR02000229[413757-416819] GCAAGTGCTGCACGTCGAAG|ATATGGTAGTAGTGTGACAG
BADR02003008.[43378-45544] CGAGTTCACGACAATGATAG|AAAGCATATATACATTACTC
BADR02002916[208288-206231] GGACCTGACCAAACCATCGG|ATGATTAAAACTGCCACTAC
BADR02000217[365838-363829] TGGTCCCAACAAGAGTCACA|TAGACGAATCACCATACGGA
