# Supplementary figures and images for: The Wide Distribution and Change of Target Specificity of R2 Non-LTR Retrotransposons in Animals
Source: PLoS One. 2016 Sep 23;11(9):e0163496. doi: 10.1371/journal.pone.0163496 (PMC5035012; doi:10.1371/journal.pone.0163496)

S2 Fig.

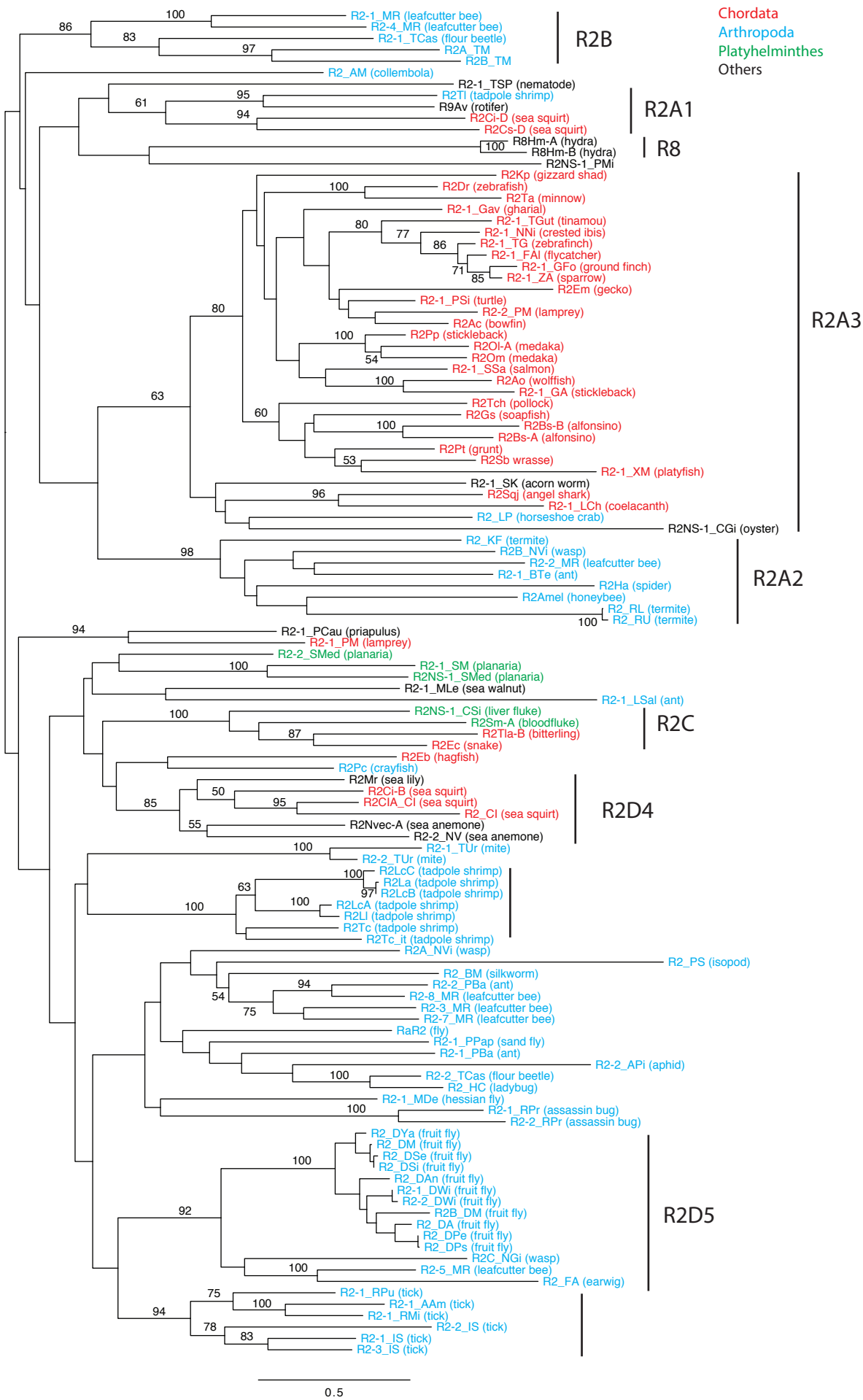

Supplement: S2 Fig — Bootstrap values above 50% are shown at branches. R2 family names and their origins are shown at leaves. R2 families from Chordata are colored in red, those from Arthropods in blue, those from Platyhelminthes in green and those from other animals in black. Clusters of R2 families that can be assigned to reported subclades are indicated by vertical lines with names and clusters not assigned to reported subclades are indicated by vertical lines but without names. (PDF) [file pone.0163496.s002.pdf]
