# Supplementary material for: The Wide Distribution and Change of Target Specificity of R2 Non-LTR Retrotransposons in Animals
Source: PLoS One. 2016 Sep 23;11(9):e0163496. doi: 10.1371/journal.pone.0163496 (PMC5035012; doi:10.1371/journal.pone.0163496)
Supplement: S3 Fig — Nucleotides identical to the 28S rRNA genes from humans are shown by dots (.). (PDF) [file pone.0163496.s003.pdf]

S3 Fig

H. sapiens 28SrDNA	TCTCTTAAGGTAGCCAAATGCCTCGTCATCTAATTAGTGACGCGCATGAATGGATGAACGAGATTCCCACTGTCCCTACCTACTATCCAG
M. musculus 28SrDNA	....
X. laevis 28SrDNA	....T..
D. rerio 28SrDNA	....G.....T..
R2Tla-B	A.GAA.TTAC...G.....T..
R2Ec	AAA..GTTAA...T..
S. mansoni 28SrDNA	....T......................T........T..
S. japonicum 28SrRNA	....T......................T........T..
S. intercalatum 28SrDNA	....T......................T........T..
S. nasale 28SrDNA	....T......................T........T..
S. indicum 28SrDNA	....T......................T........T..
