# Supplementary material for: The Wide Distribution and Change of Target Specificity of R2 Non-LTR Retrotransposons in Animals
Source: PLoS One. 2016 Sep 23;11(9):e0163496. doi: 10.1371/journal.pone.0163496 (PMC5035012; doi:10.1371/journal.pone.0163496)
Supplement: S1 Table — (PDF) [file pone.0163496.s004.pdf]

S1 Table

Class	Order / Suborder	Species	R2	Accession no.
Chondrichthyes	Squatiniformes	*Squatina japonica*	R2Sqj	LC155021
Actinopterygii	Perciformes / Percoidei	*Parapristipoma trilineatum*	R2Pt	LC155022
		Acanthopagrus latus		
		Grammistes sexlineatus	R2Gs	LC155026
		Decapterus maruadsi		
	Perciformes / Zoarcoidei	*Anarhichas orientalis*	R2Ao	LC155032
	Perciformes / Scombroidei	*Auxis rochei*		
	Perciformes / Stromateoidei	*Hyperoglyphe japonica*		
	Perciformes / Gobioidei	*Oxyurichthys papuensis*	R2Op	LC155035
	Perciformes / Labroidei	*Stethojulis bandanesis*	R2Sb	LC155038, LC155039
	Perciformes / Acanthuroidei	*Siganus vulpinus*		
		Siganus argenteus		
	Pleuronectiformes	*Pleuronichthys cornutus*		
	Lophiiformes	*Lophius litulon*		
	Tetraodontiformes	*Lagocephalus wheeleri*		
	Gasterosteiformes	*Pungitius pungitius pungitius*	R2Pp	LC155023
		Gasterosteus aculeatus		
	Beloniformes	*Oryzias melastigma*	R2Om	LC155024
		Oryzias luzonensis		
		Oryzias curvinotus		
		Oryzias celebensis		
		Tylosurus crocodilus crocodilus	R2Tcc	LC155034
	Beryciformes	*Beryx splendens*	R2BsA<br>R2BsB	LC155030<br>LC155029
	Zeiformes	*Parazen pacificus*		
	Gadiformes	*Theragra chalcogramma*	R2Tch	LC155020
	Stomiiformes	*Borostomias elucens*		

	Cypriniformes	*Rhodeus atremius atremius*	R2Raa	LC155033
		Tanakia lanceolata	R2Tla-B	LC155019
		Danio albolineatus		
	Scorpaeniformes	*Crystallichthys matsushimae*	R2Cm	LC155036
	Clupeiformes	*Konosirus punctatus*	R2Kp	LC155025
	Anguilliformes	*Anguilla marmorata*		
	Amiiformes	*Amia calva*	R2Ac	LC155031
	Acipenseriformes	*Acipenser ruthenus*	R2Ar	LC155037
Amphibia	Anura	*Xenopus mulleri*		
		Xenopus borealis		
		Xenopus laevis		
		Xenopus tropicalis		
		Rana nigromaculate		
		Bufo garagarizans		
	Caudata	*Cynops pyrrhogaster*		
Reptilia	Squamata	*Elaphe climacophora*	R2Ec	LC155028
		Eublepharis macularius	R2Em	LC155027
		Eumeces latiscutatus		
Mammalia	Monotremata	*Ornithorhynchus anatinus*		
	Didelphimorphia	*Monodelphis domestica*		
	Proboscidea	*Loxodonta africana*		
	Afrosoricida	*Echinops telfairi*		
	Cingulata	*Dasypus novemcinctus*		
	Cetartiodactyla	*Tursiops truncatus*		
	Chiroptera	*Pteropus vampyrus*		
		Myotis lucifugus		
	Eulipotyphla	*Sorex araneus*		
	Primates	*Microcebus murinus*		
		Tarsius syrichta		
	Scandentia	*Tupaia belangeri*		
